# The radiographic assessments of spino-pelvic compensation using IoT-based real-time ischial pressure adjustment

**DOI:** 10.1097/MD.0000000000028783

**Published:** 2022-02-04

**Authors:** Moon-Jun Sohn, Haenghwa Lee, Byung-Jou Lee, Hae-Won Koo, Kwang Hyeon Kim, Sang-Won Yoon

**Affiliations:** Department of Neurosurgery, Neuroscience and Radiosurgery Hybrid Research Center, Inje University Ilsan Paik Hospital, College of Medicine, Goyang, Korea.

**Keywords:** buttock, postural balance, pressure monitoring, radiographic measurement, sitting position, spinal curvature

## Abstract

In malalignment syndrome, the spino-pelvic alignment correction with foot orthotics can be applied only to a standing position in the coronal plane. Considering the fact that the average time Koreans spend sitting in a chair is 7.5 hours per day, studies on spino-pelvic correction in sitting position is needed. The purpose of this study is to investigate the pressure changes and radiographic assessment of spino-pelvic alignment using a chair equipped with a height-adjustable seat-plate. This study was conducted on 30 participants with spinopelvic malalignment. All participants were subjected to measure buttocks interface pressure while seated using a smart chair in three consecutive steps:

1. on initial seated,
2. on balancing seated, and then
3. on 1 hour balancing seated.

Radiographically, the five spino-pelvic parameters such as shoulder height differences (SHD), iliac crest height differences (ICHD), leg length discrepancy (LLD), pelvic oblique angle (POA), and coronal imbalance were analyzed to investigate the effect of pelvic imbalance compensation on spino-pelvic alignment. Statistical analysis was performed using ANOVA and paired *t* test. The pressure discrepancy improvement between buttocks from 36.4 ± 32.3 mm on initial seated to 15.7 ± 20.3 mm on balancing, 12.7 ± 10.9 mm on 1hr balancing seated (Ω, *P* *=* *.008*). The radiographic results of pelvic imbalance compensation during seated show a statistical improvement of average SHD (from −0.9 to −0.8 mm, *P* = .005) and average ICHD (from 9.5 to 2.5 mm, *P* = .037). For a standing posture after use of smart chair, average SHD value (−3.0 to −1.0 mm, *P* = .005), ICHD (from 1.8 to 0.8 mm, *P* = .016), and average LLD value (0.8–0.1 mm, *P* = .033) were statistically significant improved.

Spine-pelvic malalignment can be improved by individually customized pelvic compensation using balanced seat plate height adjustments under the real-time pressure sensing and monitoring on the buttocks while seated.

## 1 Introduction

Malalignment syndrome was defined by Wolf Schamberger, as follows: malalignment associated biomechanical changes, especially a shift in weight bearing and asymmetries of muscle tension and strength, and joint ranges of motion affecting soft tissues, joints, and organ systems throughout the body.^[1]^ The pelvis transfers loads generated by body weight and gravity during standing, walking and sitting and acts as a basis for the axial system.^[2,3]^ According to Schamberger et al there are three common presentations of pelvic malalignment; rotational malalignment, upslip of the sacroiliac joint, and inflare/outflare. Pelvic alignment influences spinal posture and stability, and pelvic malalignment is a common cause of lower back, hip, and leg pain. The symptoms and signs of malalignment syndrome include persistent foot, leg, or low back pain, change of the spine curvature, asymmetrical muscle bulk, or strength or inability to turn the body as much in a particular direction. Malalignment syndrome is commonly treated using biomechanical foot orthosis (BFO). It is the only form of therapy that addresses the correction of biomechanical malalignments in the lower extremity kinetic chain. The goal of treatment is the restoration of normal structure and function of the spine and pelvis; therefore, the devices used are designed to realign the body.^[4]^ However, the biomechanical effects of the orthoses used for the clinical treatment of malalignment syndrome are not completely understood. Previous studies have focused primarily on the effects of orthotic devices on foot structure rather than on the pelvis or spine in correction of malalignment syndrome. This study investigated the effect of IoT-based smart chair equipped with adjustable seat plate, pressure sensing and monitoring on spino-pelvic alignment and posture balance in the malalignment syndrome while seated.

## 2 Materials and methods

### 2.1 Patients

**Figure 1:**
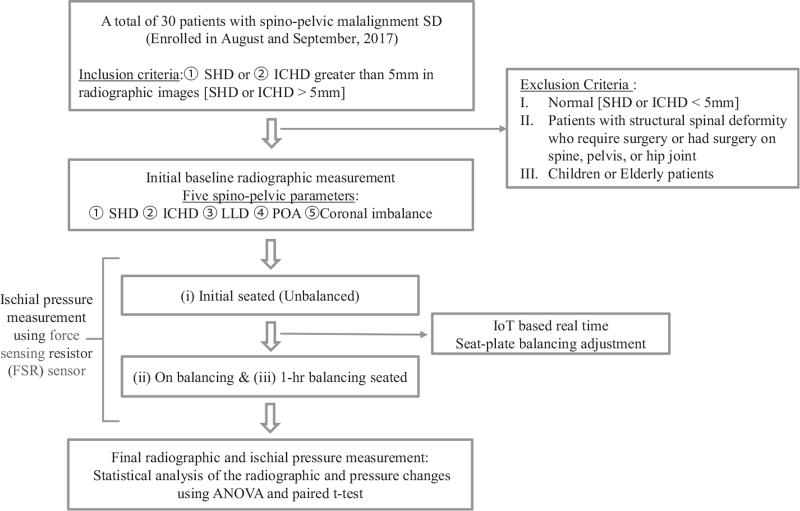
Research flow chart of the study. ICHD = Iliac crest height difference, LLD = Leg length Difference, POA = Pelvic Oblique Angle, SHD = Shoulder height difference.

From august 2017 to September 2017, a total of 30 patients with malalignment syndrome were consecutively recruited at the single institute. Diagnostic criteria of malalignment syndrome is defined as biomechanical changes, signs and symptoms (persistent foot, leg, or low back pain) consistently seen in association with 2 of these indications: rotational malalignment, upslip of the sacroiliac joint, and inflare/outflare.^[1]^ All patients provided informed consent, and the protocols involving baseline measures and contact for re-measurement were approved by the institutional review board (No 2017-07-010-006). The inclusion criteria were as follows: The participants of nonstructural malignment syndrome with shoulder height differences (SHD) or iliac crest height differences (ICHD) >5 mm in radiographic images excluding participants with structural deformity. The participants within a normal range with SHD or ICHD of <5 mm are excluded for the qualitative level of the study in considering measuring errors. The exclusion criteria were as follows: patients with structural spinal deformity or who require surgery or had surgery on spine, pelvis, or hip joint, and children or elderly persons. All patients with eligible inclusion criteria were measured for baseline value. This number of patients exceeds the minimum number of cases per variable (of at least two) for five independent variables in our linear regression models.^[5]^ The 30 participants consist of 21 females and 9 males. In the 21 female participants, the average age was 27 ± 7 years old (19 old to 58 years old), the average height was 161.9 ± 5.6 cm (150–173 cm), and the average weight was 55 ± 9.5 kg (42–75 kg). In the 9 male participants, the average age was 36 ± 12.1 old (19–50 years old), the average height was 173 ± 4.8 cm (167–182 cm), and the average weight was 70 ± 14.4 kg (50–98 kg). A research flow chart for this study of spino-pelvic compensation using IoT-based real-time ischial pressure adjustment is shown in Figure 1. Initially, baseline radiographic measurement was conducted for the enrolled 30 participants as in the following radiographic assessment. Then, individual patient's initial unbalanced ischial pressure on seated was measured from force-sensitive resistor (FSR) sensors and followed by balanced ischial pressure was evaluated on balancing and 1hour balancing seated under IoT-based real-time seat plate adjustments. Finally, the values of the difference in seat interface pressure between the left and right buttocks in the three steps:

1. on initial seated,
2. on balancing seated, and
3. on 1-hour balancing seated were compared.

Changes in radiographic vertebrae parameters in sitting and standing positions were also compared statistically on initial unbalanced seating and an hour-balancing seated.

### 2.2 Radiographic assessment

**Figure 2:**
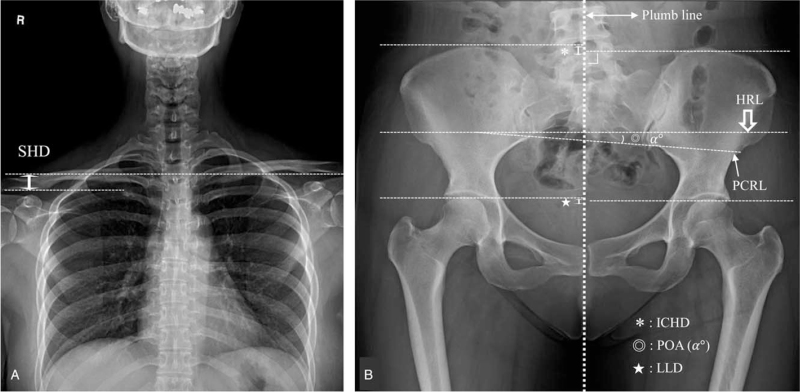
Evaluation of radiographic parameters for spinopelvic imbalance measurement on coronal image. The radiographic parameters included (A) shoulder height differences (SHD, double big arrow), (B) iliac crest height differences (ICHD, asterix), leg length discrepancy (LLD, star), pelvic oblique angle (POA) indicates angle (double circle, @) between horizontal line (HRL, big open arrow) and pelvic coronal reference line (PCRL, narrow arrow).

The anteroposterior x-ray images were obtained in the sitting and standing positions. The five spino-pelvic parameters for evaluation of smart chair were as follows: SHD, ICHD, leg length discrepancy (LLD), pelvic oblique angle (POA), and coronal imbalance (Fig. 2). SHD was measured as the height difference of the soft tissue shadows directly superior to the acromioclavicular joints (positive is defined as left shoulder up/right shoulder down).^[6]^ ICHD and LLD values were defined as the vertical distance between 2 lines when the horizontal line is drawn from the superior aspect of the iliac crest and femoral head to the plumb line with the right angle in the anterior-posterior radiograph of the pelvis.^[7]^ POA was defined as the angle between first line (horizontal line, HRL) and second line (pelvic coronal reference line, PCRL).^[8]^ Coronal imbalance was defined as a >20 mm distance between the C7 plumb line and central sacral vertical line (CSVL). It was defined as positive imbalance when the C7 plumb line passes 2 cm or more to the right side of CSVL, and negative imbalance when C7 plumb line passes 2 cm or more to the left.^[9]^

### 2.3 Seat-interface pressure monitoring and balanced adjustment using smart chair

**Figure 3:**
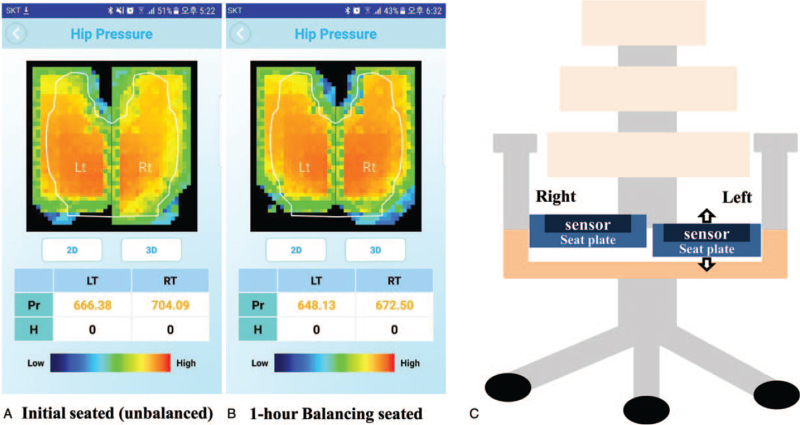
Measurement of ischial pressure using a smart chair. User experience/user interface (UX/UI) display image for ischial pressure measurement (A and B). Initial unbalanced pressure (A) was 666.38 to 704.09, a difference of 37.71, followed by a 1 mm lower left seat plate for balancing, and improved pressure changes (B) during 1-h balanced seating (648.13 vs 672.50, a difference of 24.37). (C) Diagram of the smart chair using adjustable seat plate equipped with pressure sensor. Only left seat plate is adjustable height for balanced pressure distribution.

Smart chair (patent No. US 10,194,754 B2) was used to measure the pressure of the buttocks in the patient's sitting position, and to balance both buttocks’ pressures by the left seat plate equipped with adjustable height motor (Fig. 3). We analyzed the seat-interface pressure for three steps:

1. on initial seated,
2. on balancing seated, and
3. on 1hr balancing seated.

The pressure sensors that installed on each seat plates measure the seat-interface pressure applied to both the buttocks while sitting on smart chair. Then, the measured results are displayed on a mobile internet of things (IOT) device's user experience/user interface (UX/UI). Balance adjustment was performed by adjusting the height of the left seat plate; When left pressure was large, height of the left seat plate was increased (positive value adjustment). When right pressure was large, height of the left seat was lower (negative value adjustment). When adjusting the height of the left seat plate to balance both pressures, the seat-interface pressures were measured. After 1hr balancing seated, the pressure was measured again.

### 2.4 Research flow process

The research was conducted on 30 research participants diagnosed with malalignment syndrome, as previously defined by the Patients section. The seat-interface pressure measurements and radiographic assessments of all participants are as follows (Fig. 1).

1. In radiographic images of all participants at the sitting and standing positions, the radiographic assessments were investigated.
2. Smart chair measured seat-interface pressure by both ischium on the parallel seat plates. The height of the left seat is adjusted to balance the left and right pressures, and then seat-interface pressures were obtained again.
3. The seat-interface pressure values of all participants were obtained after 1hr balancing seated. The radiographic assessments using x-ray images at the sitting and standing positions are measured and analyzed.

### 2.5 Statistical analysis

The values measured by 2 observers with more than 5 years of experience in the spine field were independently used. Intra-examiner reproducibility and inter-examiner reliability were examined using the intra- and inter-class correlation coefficients. Unbalance and balanced pressure measurements were analyzed using ANOVA test and paired *t* test. All analyses were conducted using Prism version 7 (Graphpad, San Diego).

## 3 Results

### 3.1 Measured seat-interface pressure before/after use of smart chair

**Figure 4:**
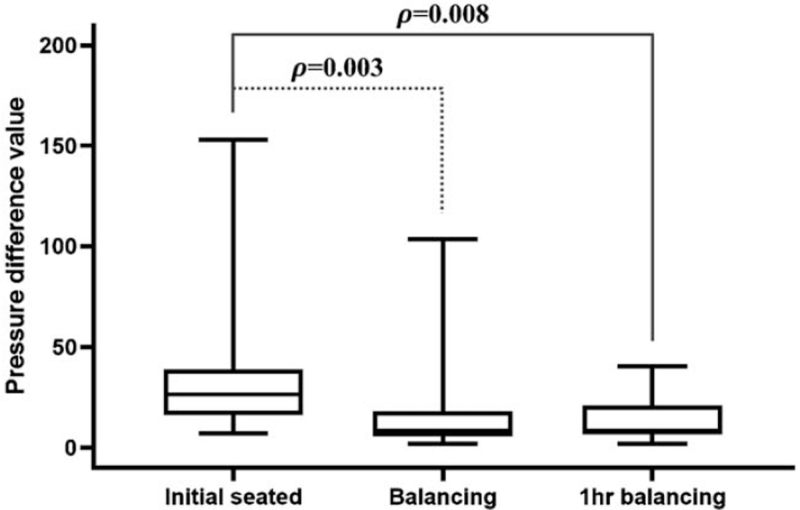
Comparison of difference values in seat-interface pressure. Seat-interface pressure between left and right buttock measured from the three steps: on initial seated, on balancing seated, and on 1 h balancing seated. The average pressure difference value was 36.4 ± 32.3 Ω for initial seating, 15.7 ± 20.3 Ω on subsequent balanced seating, and 12.7 ± 10.9 Ω on 1 h balancing seated, demonstrating statistically significant improvements among the steps (*P* = .003, .008, ANOVA).

We analyzed difference values of seat-interface pressure between left and right buttock for three steps:

1. initial seated,
2. on balancing seated, and
3. on 1hr balancing seated (Fig. 4).

For the initial unbalanced seating, the average pressure difference value was 36.4 ± 32.3 Ω (7.0–153.1 Ω). The average height of left seat was changed by 3.7 ± 1.7 mm (1–8 mm) to balance left and right pressure, and the average pressure difference was 15.7 ± 20.3 Ω (1.8–103.4 Ω, *P* *=* *.003*) on balancing seated. The average difference values after 1 hour balancing seated was 12.7 ± 10.9 Ω (1.8–40.4 Ω, *P* *=* *.008*). These results show that the pressure difference measured after an hour was improved by 65.1%.

### 3.2 Spino-pelvic alignment factors in sitting position and standing position

The spino-pelvic alignment results at initial and after 1hr balancing seated are summarized in Table 1. Both the sitting and standing postures of patient were evaluated. In the initial seated with the seat plate parallel (unbalanced), average SHD was −0.9 ± 7.1 mm (−15.8 to 12.3 mm), and average ICHD value was were 9.5 ± 10.0 mm (−11.3 to 35.5 mm). After 1 hour balancing seated, average SHD and average ICHD for sitting position were −0.8 ± 6.6 mm (−18.6 to 6.8 mm) and 2.5 ± 10.4 mm (−24.2 to 21.1 mm), respectively. For the participants in the standing position at initial seated, average values of SHD, ICHD, LLD, POA, and coronal imbalance were −3.0 ± 7.1 mm (−17.4 to 17 mm), 1.8 ± 8.5 mm (−16.5 to 16.4 mm), 0.8 ± 7.6 mm (−16.8 to 13.4 mm), 0.5 ± 3.0° (−6.8° to 5.0°), and 3.5 ± 8.2 mm (−11.8 to 21.6 mm), respectively. After balancing seated for 1hr, average SHD, average ICHD value, average LLD, average POA, and average coronal imbalance for standing position were −1.0 ± 6.3 mm (−14.2 to 13 mm), 0.8 ± 7.4 mm (−14.4 to 14.3 mm), 0.1 ± 6.9 mm (−16.5 to 13.4 mm), 0.3 ± 2.7° (−6.0° to 4.0°), and 3.4 ± 6.9 mm (−10.0 to 18.7 mm), respectively.

**Table 1:** Malalignment change results in sitting position and standing position at initial and after 1hr balancing seated.

		Average value			
Position	Measurement of radiographic parameters	On initial seated	1 h balancing seated	Improvement (%)	*P*
Sitting	SHD (mm)	−0.9 ± 7.1	−0.8 ± 6.6	11.1	.005
	ICHD (mm)	9.5 ± 10.0	2.5 ± 10.4	73.7	.037
Standing	SHD (mm)	−3.0 ± 7.1	−1.0 ± 6.3	66.7	.005
	ICHD (mm)	1.8 ± 8.5	0.8 ± 7.4	55.6	.016
	LLD (mm)	0.8 ± 7.6	0.1 ± 6.9	87.5	.033
	POA (°)	0.5 ± 3.0	0.3 ± 2.7	40.0	.017
	Coronal imbalance (mm)	3.5 ± 8.2	3.4 ± 6.9	2.9	.040

ICHD = iliac crest height differences, LLD = leg length discrepancy, POA = pelvic oblique angle, SHD = shoulder height differences.

**Figure 5:**
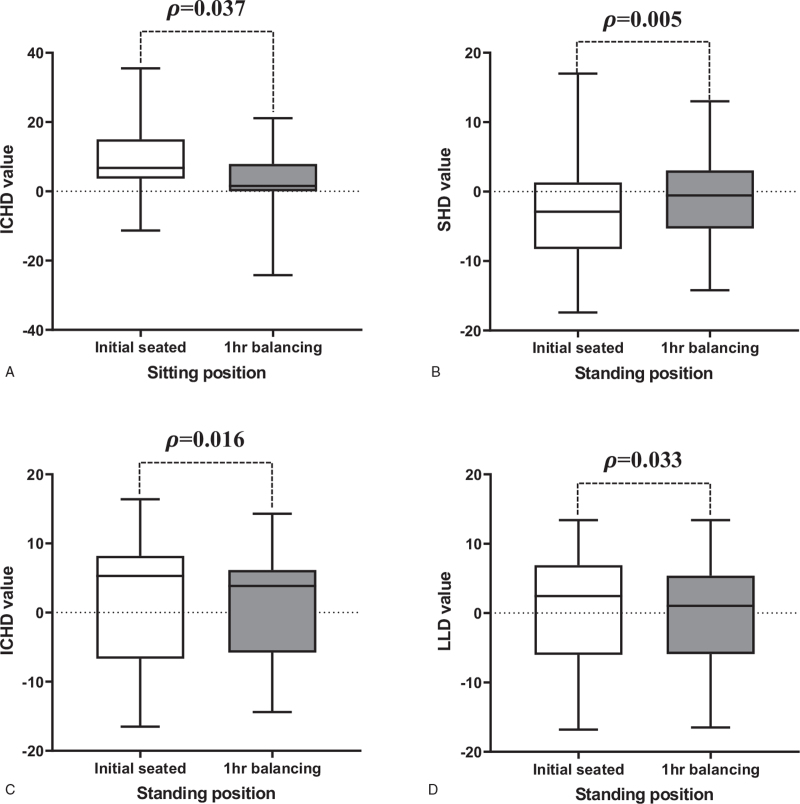
Comparison of changes in the spinopelvic parameters. The changes of the spino-pelvic parameters in sitting and standing position were compared at initial unbalanced seating and after 1hr balancing seated. In sitting position, the average ICHD value (A) was statistically significant improvement from 9.5 ± 10.0 mm at initial seating to 2.5 ± 10.4 mm for 1 h balancing seated (*P* *=* *.037*). The standing position also showed statistically improvements with average SHD value (−3.0 to −1.0 mm, *P* *=* *.005*), average ICHD value (1.8–0.8 mm, *P* *=* *.016*) and average LLD value (0.8–0.1 mm, *P* *=* *.033*) by paired *t* test (B–D, respectively).

**Figure 6:**
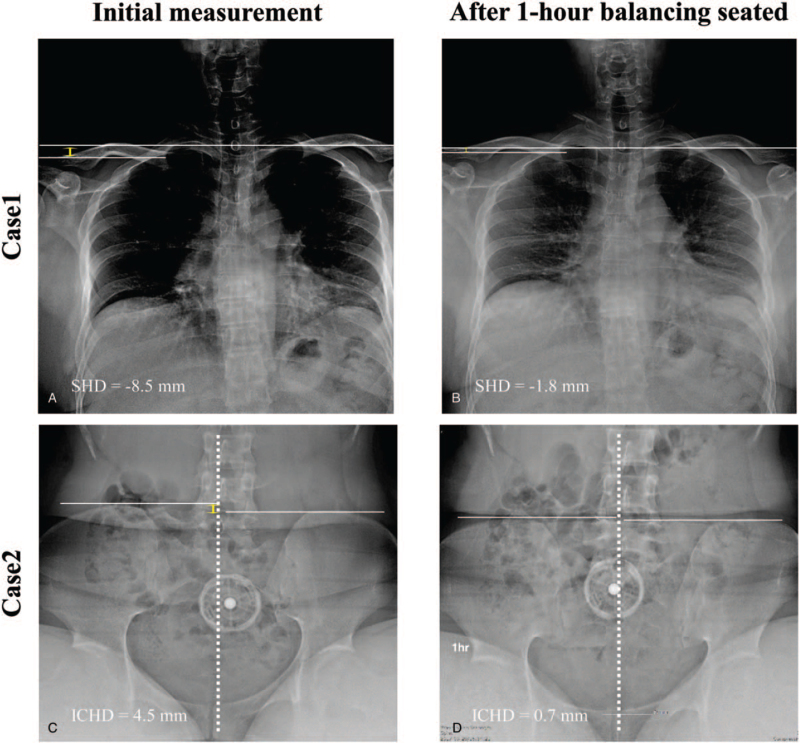
Case illustration of changes observed in the measurement results of radiographic parameters. Changes of spinopelvic parameters were observed in the radiographic measurement of initial unbalanced seating and 1 h balancing seated. The SHD value was improved from −8.5 mm (A) on initial seated to −1.8 mm (B) on 1 h balancing seated (case 1). In case 2, the measurement of the ICHD value improved from 4.5 mm (C) to 0.7 mm (D) during 1hr balancing seated.

The improvement of all the above measurements was statistically significant (Fig. 5). In sitting position, average ICHD improved from 9.5 to 2.5 mm (*P* *=* *.037*), and was statistically significant. In standing position, average values of SHD value (−3.0 to −1.0 mm, *P* *=* *.005*), ICHD (1.8–0.8 mm, *P* *=* *.016*), and LLD (0.8–0.1 mm, *P* *=* *.033*) improved. Figure 6 shows spino-pelvic parameters of two cases at initial and after 1 hour balancing seated. In these results, SHD (−8.5 to −1.8 mm, 78.8%) and ICHD (from 4.5 to 0.7 mm, 80.8%) values improved by balanced sitting for 1 hour.

## 4 Discussion

The term “malalignment syndrome” is considered to be a biomechanical change accompanied by signs associated with rotational malalignment of pelvis, upslip of the sacroiliac joint, and inflare/outflare of pelvis surfaces.^[1]^ Abnormal loading to pelvis that exceed this normal displacement in any direction can cause the adjoining SI joint surfaces to end up in an aberrant position so that the surfaces no longer match and stay compressed in some areas, separated in others, affecting normal movement.^[10]^ If the surfaces of SI joint become fixed or locked in an abnormal position, major consequences include dysfunction of SI joint mobility, a disturbance of the lumbo–pelvic–hip complex and its ability to transfer weight and absorb shock, and an alteration of gait.^[1]^ Deformation and degenerative changes in spinal posture cause changes in the coronal and sagittal plane arrangement and make the changes in the whole spine alignment and deformation.^[1,11,12]^ The arrangement of the coronal and sagittal plane involves compensatory mechanism using the change of the lower limbs and the balance of the spine-pelvis.^[13,14]^ Change of the spinal-pelvic balance is the starting point for inducing a compensatory response that accounts for the entire spinal imbalance.^[13,15]^ Therefore, the compensatory response of the pelvis occurs simultaneously with the change of the legs as the imbalance increases. The effect of compensatory mechanism is appeared more prevalent in the pelvis and lower extremities, and the adaptive response in the thoracic alignment is less.^[14,16]^ However, this compensatory mechanism is also related age. In the course of “natural cascade of degeneration,” the elderly secures the stability of the spine by restabilization process including facet joint hypertrophy, bony overgrowth, and ossification of the anterior and posterior longitudinal ligament. Therefore, the older the age, it is harder to correct the spinal-pelvic imbalance by this compensatory mechanism.^[16]^ The younger the age, the faster correction of spinal pelvic imbalance is needed.

Malalignment syndrome is commonly treated using BFO treatment, the only therapy that corrects biomechanical malalignments in the lower extremity kinetic chain. The goal of this method is to restore normal structure and function of the spine and pelvis; therefore, the devices used are designed to realign the body. These orthotics increase foot stability by providing contact for weight bearing across a larger part of the sole and decrease the tendency of the feet to roll inwards or outwards after alignment has been achieved, and may decrease torque forces on legs.^[17]^ Orthotics increase sensory input from the sole surface, and the stimulation of proprioceptive receptors has been shown to help control pain. A reduced perception of pain may also elicit reflex relaxation of muscles, which may help to reduce muscle tension asymmetry.^[4,18]^ Orthotic intervention is believed to influence the pattern of lower extremity movement through a combination of mechanical control and biofeedback.^[4,19]^ Kim et al found that BFO reduce pelvic-tilt sidedness, and pelvic rotation angle asymmetry was significantly decreased in the BFO condition, compared to the barefoot and shoes conditions, which implies that BFO contributes to the correction of pelvic rotational asymmetry.^[4]^ However, this study participant had moderate to severe pelvic asymmetry, and it remains undetermined whether BFO is effective for patients with mild pelvic asymmetry.^[19]^

The starting point of the compensatory mechanism of spinal imbalance is the change of pelvis and leg position. The foot orthosis can correct the pelvis asymmetry by change the leg position. However, there is no way to correct the pelvis asymmetry by acting directly on the pelvis. Therefore, we developed a smart chair that could directly correct the imbalance of the pelvis by directly affecting the pelvic and evaluated its effectiveness. The present study described the immediate influence of smart chair on malalignment syndrome, especially with respect to change in pelvic asymmetry. This study found that pelvic asymmetry was significant decreased in the smart chair condition, which implies that smart chair contributes to the correction of pelvic asymmetry.

Kim et al^[4]^ reported the result of improved pelvic tilt of 0.92 ± 0.42 using biomechanical foot orthosis (BFO) and shoes (Table 2). In addition, although foot orthoses were used in several studies,^[18,19]^ we obtained an improved discrepancy reduction using our IoT-based real-time pressure sensing and monitoring on the buttocks with a smart chair.

**Table 2:** Comparison of studies related to balance compensation.

Authors (year)	Patients (n)	Method	Summary
Kim et al^[4]^ (2016)	10	Biomechanical foot orthosis (BFO) and shoes	BFO can correct pelvic asymmetry while walking (peak pelvic tilt and obliquity angle were significantly greater (*P* = .037, .02).
Lee JG et al^[19]^ (2018)	52	Retrospective study for the patients with prescribed custom molded foot orthosis (FO) to correct in equality of RCSPA (Resting calcaneal stance position angle)	By wearing FO, Cobb angle was improved from 22.03° ± 4.30° to 18.86° ± 7.53° (most in 9 months) Pelvic height and RCSPA difference were reduced from 1.07 ± 0.25 cm to 0.6 ± 0.36 cm and from 4.25° ± 0.71° to 1.71° ± 0.75°, respectively (*P* < .01). FO is useful in correcting functional factors; pelvic inequality
This study (present)	30	IoT-based real-time ischial pressure monitoring and adjustment for balanced seating	Balanced sitting improved 65.1% of Ischial pressure difference. Radiographic spinopelvic parameters; In sitting, improvement in average value of ICHD (from 9.5 to 2.5 mm, *P* *=* *.037*). In standing, improvement in average values of SHD (−3.0 to −1.0 mm, *P* *=* *.005*), ICHD (1.8–0.8 mm, *P* *=* *.016*), and LLD (0.8–0.1 mm, *P* *=* *.033*), respectively by balanced sitting for 1 h.

BFO = biomechanical foot orthosis, FO = foot orthosis, ICHD = Iliac crest height difference, LLD = Leg length difference, RCSPA = resting calcaneal stance position angle, SHD = shoulder height difference.

The shortcomings of this study are as follows. First, there is limited number of enrolled cases in this study. The main reason for this small number of registrations is that the awareness of spino-pelvic imbalance in the coronary plane was ambiguous, although it is well recognized that the functional imbalance of the spine progresses and eventually fixes to structural deformation. Thus, in order to determine the inclusion criteria, clear exclusion criteria were first established, such as structurally fixed spinal deformity patients. Relatively younger groups were registered in this study whilst the elderly groups with structural changes in the natural aging process were excluded. The elderly patient with severe or irreversible degenerative structural change is more likely to be unresponsive to the alignment correction using the smart chair. Second, only coronal radiographic parameters measured is available at the sitting position in this study. Generally, most of the radiographic parameters are evaluated in sagittal plan and or standing position. Lastly, the appropriate time of the intervention applying the smart chair was not established yet. Therefore, additional studies are needed to confirm whether 1 hour apply is really effective in correcting the alignment. The study of the degree of effect of applying over time is essential later. Despite these limitations, the importance of this study was conducted to understand the effect of intervening in functional changes in daily life so that functional spinal deformation does not progress to structural deformation.

## 5 Conclusion

Spine-pelvic malalignment can be improved by individually customized pelvic compensation using balanced seat plate height adjustments under the IoT-based real-time pressure sensing and monitoring on the buttocks while seated.

## Author contributions

M-J. S. conceived of the presented idea and supervised the project. H-W. K., K. H. K., S-W. Y carried out the experiment. H. L. performed the analytic calculation. M-J. S. took the lead in writing the manuscript. H-W. K., K. H. K., and S-W. Y contributed to the interpretation of the results. M-J. S. and H. L. contributed to the final version of the manuscript. All authors provided critical feedback and helped shape the research, analysis and manuscript.

**Data curation:** Haenghwa Lee.

**Investigation:** Byung-Jou Lee, Hae-Won Koo, Kwang Hyeon Kim, Sang-Won Yoon.

**Methodology:** Hae-Won Koo, Kwang Hyeon Kim, Sang-Won Yoon.

**Project administration:** Moon-Jun Sohn, Byung-Jou Lee.

**Supervision:** Moon-Jun Sohn.

**Validation:** Hae-Won Koo, Kwang Hyeon Kim, Sang-Won Yoon.

**Writing – original draft:** Moon-Jun Sohn, Byung-Jou Lee.

**Writing – review & editing:** Moon-Jun Sohn, Haenghwa Lee.
